# Astrocyte–blood–brain barrier crosstalk in cerebral small vessel disease: linking barrier dysfunction to neurovascular failure

**DOI:** 10.3389/fneur.2026.1895597

**Published:** 2026-08-05

**Authors:** Mei Xie, Shuang Wu, Ling Yang, Yuqiang Zhang

**Affiliations:** 1 Department of Rehabilitation and Pain Medicine, Ziyang Central Hospital, Ziyang, China; 2 Department of Anesthesiology, Ziyang Central Hospital, Ziyang, China

**Keywords:** astrocytes, blood–brain barrier, cerebral small vessel disease, neurovascular coupling, neurovascular unit

## Abstract

Cerebral small vessel disease (CSVD) is a heterogeneous group of disorders involving the cerebral microvasculature and is a major cause of stroke and vascular cognitive impairment. The role of blood–brain barrier (BBB) dysfunction in CSVD is now recognized as a key pathological process; however, conventional vascular risk factors do not fully explain its initiation, progression, or clinical heterogeneity. Astrocyte–BBB crosstalk has been implicated in endothelial dysfunction, pericyte injury, and vascular remodeling, and the mechanisms by which early barrier instability progresses to neurovascular failure remain poorly understood. This review highlights astrocytes as active regulators of BBB integrity and neurovascular unit homeostasis. Astrocytic endfeet support endothelial junctions, regulate basement membrane structure, and maintain water and ion homeostasis, perivascular exchange, inflammatory regulation, and neurovascular coupling. Astrocytes may shift from a homeostatic to a reactive state following chronic hypoperfusion, inflammatory stress, metabolic injury, or APOE4-related vulnerability, leading to increased inflammatory signaling, loss of AQP4 and Kir4.1 polarization, basement membrane remodeling, and pericyte dysfunction. Barrier instability persists, resulting in impaired glymphatic clearance, disrupted cerebral blood flow regulation, and white matter and cognitive impairment. We hypothesize a key mechanistic link between BBB dysfunction and neurovascular failure in CSVD related to disrupted astrocyte–BBB crosstalk. However, much of the current evidence is derived from animal models and *in vitro* systems, and direct human data–particularly from longitudinal imaging and biomarker cohorts–remain limited. Future work should integrate imaging, biomarkers, and stratified therapies to improve early detection and restore astrocytic endfoot polarity and BBB homeostasis.

## 1 Introduction

Cerebral small vessel disease (CSVD) comprises a heterogeneous group of disorders arising from intrinsic injury to the cerebral microvasculature. It is commonly associated with aging, hypertension, and genetic factors, and represents a major vascular contributor to stroke and cognitive impairment, imposing a substantial global public health burden (1–3). Typical neuroimaging features of CSVD include lacunes, cerebral microbleeds, white matter hyperintensities (WMH), and enlarged perivascular spaces (PVS) (4). Epidemiological studies have shown that the prevalence of moderate-to-severe WMH can reach 20.5, 40.5, and 58.4% in community-dwelling, stroke, and dementia populations, respectively. Lacunes and cerebral microbleeds are also common in patients with stroke, and nearly 40% of patients with lacunar stroke have vascular cognitive impairment (5, 6). Although traditional cardiovascular and cerebrovascular risk factors, such as hypertension and diabetes mellitus, are strongly associated with CSVD development and progression (7, 8). CSVD-like pathological changes have also been observed in individuals without overt vascular risk factors (9–11). Recent reviews have comprehensively examined the link between hypertension and BBB disruption, highlighting the role of the neurovascular unit–including astrocytes–in this process (12). These findings suggest that classical vascular risk factors alone are insufficient to fully explain the complexity and heterogeneity of CSVD pathogenesis.

Increasing evidence indicates that CSVD progression is closely associated with dysfunction of the neurovascular unit (NVU). Impaired NVU function has been suggested to increase blood–brain barrier (BBB) permeability and is associated with CSVD-related neurological deficits (13, 14). Structurally, the BBB is primarily formed by endothelial cells and is coordinately regulated by pericytes, astrocytes, and other cellular components (15). The role of these cellular components in the context of hypertension and BBB dysfunction has been reviewed in detail elsewhere (12). As a key component of the NVU, the BBB not only restricts the entry of blood-borne constituents and potentially harmful substances into the brain parenchyma but also contributes to the clearance of metabolic waste and neurotoxic products (15, 16).

Throughout this review, we use the following terminology: “BBB dysfunction” is the overarching term for any impairment in barrier function; “BBB instability” refers to early, reversible functional alterations (e.g., increased permeability without structural damage); “BBB disruption” and “BBB injury” denote more advanced structural damage, including tight junction breakdown, basement membrane remodeling, and endothelial injury. Where the cited literature uses specific terms, we retain the original wording but apply these definitions for conceptual consistency.

Previous studies on CSVD have largely focused on pericyte injury, endothelial dysfunction, and vascular wall remodeling (17, 18). In recent years, astrocytes have been increasingly recognized as central regulators of BBB homeostasis and cerebral microvascular function (14, 19, 20). Astrocytic endfeet extensively ensheathe cerebral microvessels and regulate BBB homeostasis, playing critical roles in cerebral fluid and solute exchange as well as neuroinflammation (19–21). Under chronic pathological conditions, astrocytes undergo reactive transformation and structural remodeling, which may disrupt BBB homeostasis (22, 23), disturb the cerebral microenvironment, and ultimately contribute to the characteristic neuroimaging features and clinical manifestations of CSVD. Given that the underlying mechanisms remain incompletely defined and that disease-specific therapeutic strategies are still lacking beyond the control of vascular risk factors (7, 24), this process provides an important rationale for the present review.

This review aims to provide a narrative review on the mechanisms and potential pathological links of astrocyte–BBB crosstalk in CSVD, thereby providing a conceptual basis for future targeted diagnostic and interventional strategies. To provide an immediate overview of the conceptual framework, Table 1 summarizes the classical CSVD mechanisms alongside the astrocyte-dependent pathways proposed in this review.

**Table 1:** Classical and astrocyte-dependent mechanisms in CSVD pathogenesis.

Classical/established CSVD mechanisms	Proposed astrocyte-dependent pathways	Key mediators and experimental evidence
Endothelial dysfunction	Inflammatory barrier injury	Pro-inflammatory cytokines (IL-6, TNF-α), chemokines (CXCL1, CXCL10), NF-κB signaling; evidence from CSVD models and related neurological conditions (83–86)
Reduced NO bioavailability, impaired vascular reactivity, increased adhesiveness; well-established in human CSVD and animal models	Reactive astrocytes promote immune cell infiltration and directly damage endothelial tight junctions through secreted mediators (S100B, iNOS/NO) (56, 88)	
Pericyte injury and loss	Loss of endfoot polarity	AQP4 and Kir4.1 mislocalization from perivascular membrane domain; DAPC (dystrophin-associated protein complex) disruption; MMP9-mediated β-dystroglycan cleavage (20, 39, 91, 92, 96, 97)
Pericyte degeneration, reduced vascular coverage, impaired capillary hemodynamics; demonstrated in CSVD and APOE4 models	Reduced AQP4/Kir4.1 polarization disrupts water-ion homeostasis and weakens structural support for endothelial junctions and basement membrane (20, 96)	
Vascular wall remodeling	Endfoot–basement membrane uncoupling	TGF-β1/SMAD3 signaling, MMP9 activation, LAMC1 dysregulation; leads to basement membrane thickening, vascular stiffening, and microbleeds (41, 100, 101)
Basement membrane thickening, fibrosis, loss of vascular compliance; driven by TGF-β/SMAD pathways and MMP activation (2, 100, 101)	Physical and molecular detachment of astrocytic endfeet from the vascular basement membrane, amplifying structural BBB disruption (99)	
Chronic hypoperfusion	Glymphatic dysfunction	AQP4 polarity loss, OAP (orthogonal arrays of particles) disassembly; DTI-ALPS reduction; EPVS (enlarged perivascular spaces); impaired CSF-ISF exchange (94, 103)
Reduced cerebral blood flow, white matter ischemia, energy failure; common in aging, hypertension, and CSVD	Impaired perivascular fluid flow and metabolic waste clearance, leading to ISF stasis, EPVS, and toxic metabolite accumulation (112–115)	
Neurovascular uncoupling	Neurovascular uncoupling	Astrocytic Ca^2+^ dysregulation, imbalanced vasoactive mediators (EETs, 20-HETE, PGE2), Kir4.1 dysfunction; leads to cerebral blood flow-metabolism mismatch (117–119, 123, 125)
Impaired functional hyperemia, reduced cerebrovascular reactivity, autoregulatory failure	Astrocytes fail to translate neuronal activity into appropriate vasomotor responses due to disrupted Ca^2+^ signaling, loss of endfoot polarity, and physical endfoot detachment from vessels (121, 126)	

For this review, we conducted a comprehensive literature search in PubMed and Web of Science up to December 2025, using combinations of keywords including “cerebral small vessel disease”, “astrocyte”, “blood–brain barrier”, “neurovascular unit”, “AQP4”, “glymphatic system”, and “APOE4”. We prioritized peer-reviewed original articles, systematic reviews, and major consensus statements. Given the narrative nature of this review and the relative paucity of direct CSVD-specific mechanistic studies, we also included evidence from related neurological conditions (e.g., ischemic stroke, intracerebral hemorrhage, Alzheimer’s disease, and traumatic brain injury) where biologically relevant pathways may inform CSVD pathophysiology. These sources are explicitly identified throughout the manuscript.

## 2 Structural and functional mechanisms of astrocytes

### 2.1 Vascular-associated anatomy of astrocytes

**Figure 1:**
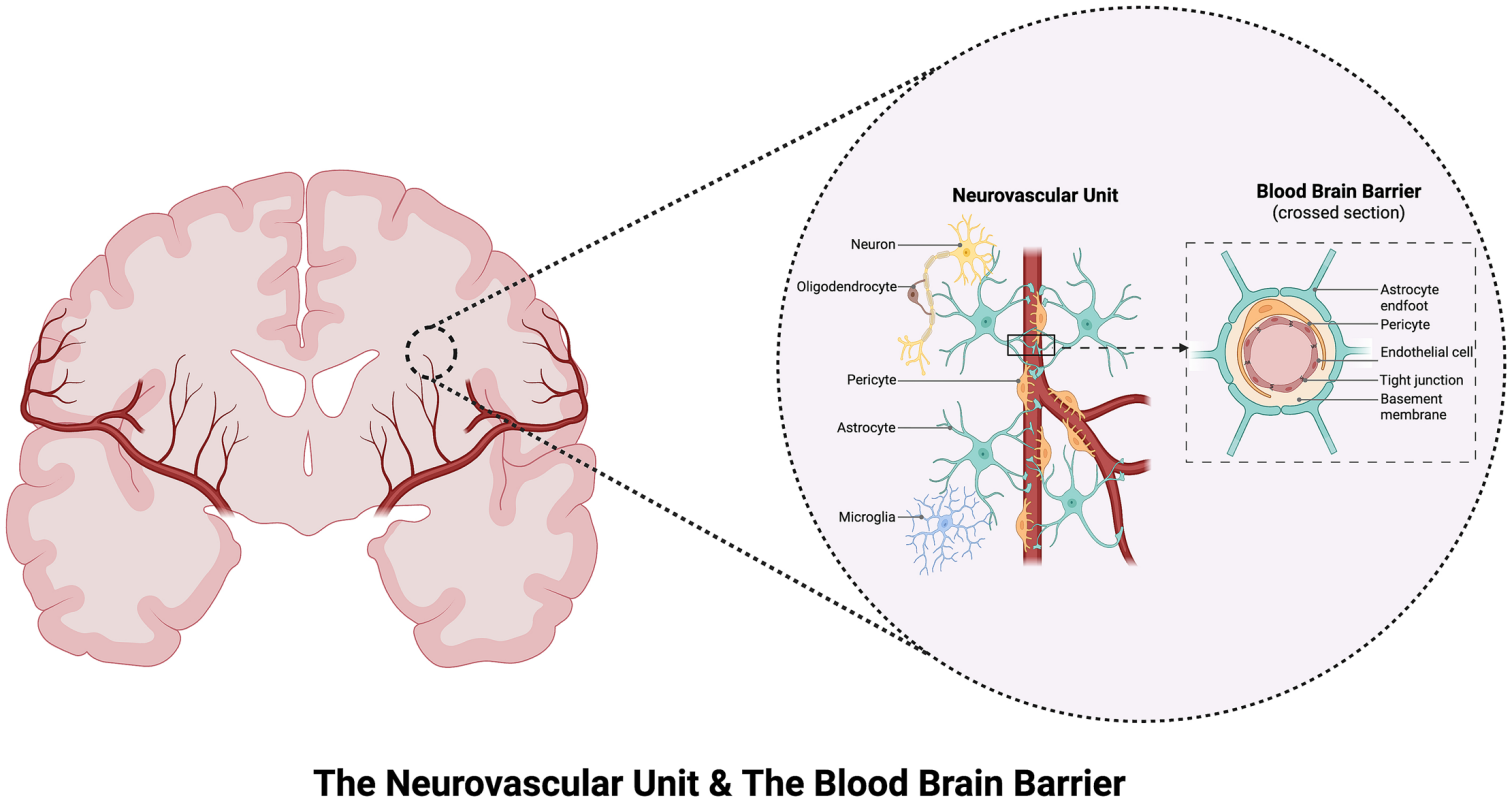
Structure of the astrocyte–centered NVU and BBB. The NVU is composed of endothelial cells, pericytes, astrocytes, and adjacent neural cells. Within this interface, astrocytic endfeet ensheath cerebral microvessels and contribute to the maintenance of BBB integrity, vascular stability, and neurovascular homeostasis. The BBB is primarily formed by tightly connected endothelial cells, the basement membrane, pericytes, and astrocytic endfeet. The molecular anchoring machinery that stabilizes astrocytic endfeet at the perivascular interface, including the dystrophin-associated protein complex (DAPC) and its interaction with basement membrane laminins, is illustrated in detail in Figure 2 (created with BioRender.com).

Astrocytes are among the most abundant glial cells in the central nervous system (CNS), accounting for approximately 20–50% of total CNS cells, and are core components of the NVU (25). Astrocytes extend multiple branched processes from the soma. Some processes directly contact neuronal synapses, whereas others project along the abluminal interface of cerebral microvessels and expand at their distal ends to form astrocytic endfeet. These endfeet ensheathe cerebral vessels and the pericyte-associated basement membrane, and together with endothelial cells constitute the perivascular interface of the BBB (20, 26, 27). Astrocytic endfeet are enriched in various channel proteins and molecular anchoring complexes, providing a key structural basis for astrocytes to sense and regulate the cerebral microvascular environment (26) (Figure 1).

This specialized spatial organization enables astrocytes not only to contribute to BBB structural stability but also to regulate neurovascular coupling (NVC), perivascular fluid exchange, and metabolic waste clearance (20, 28). Studies have demonstrated direct contacts and multiple ligand–receptor interactions between astrocytic endfeet and endothelial cells (27, 29). Thus, the vascular-associated anatomy of astrocytes provides the structural basis for astrocyte–BBB crosstalk.

### 2.2 Endfoot polarity proteins and molecular anchoring machinery

The function of astrocytic endfeet depends on the selective enrichment of multiple membrane proteins and anchoring molecules within the perivascular membrane domain, a process referred to as endfoot polarization. Endfoot polarization does not merely indicate increased expression of these proteins; rather, it emphasizes their stable, spatially directed distribution along the endfoot membrane, thereby forming a molecular organizational pattern that matches the cerebral microvascular interface (26, 30). This polarized architecture enables astrocytes to regulate BBB microenvironmental homeostasis locally and constitutes an important molecular basis for astrocyte–BBB crosstalk.

**Figure 2:**
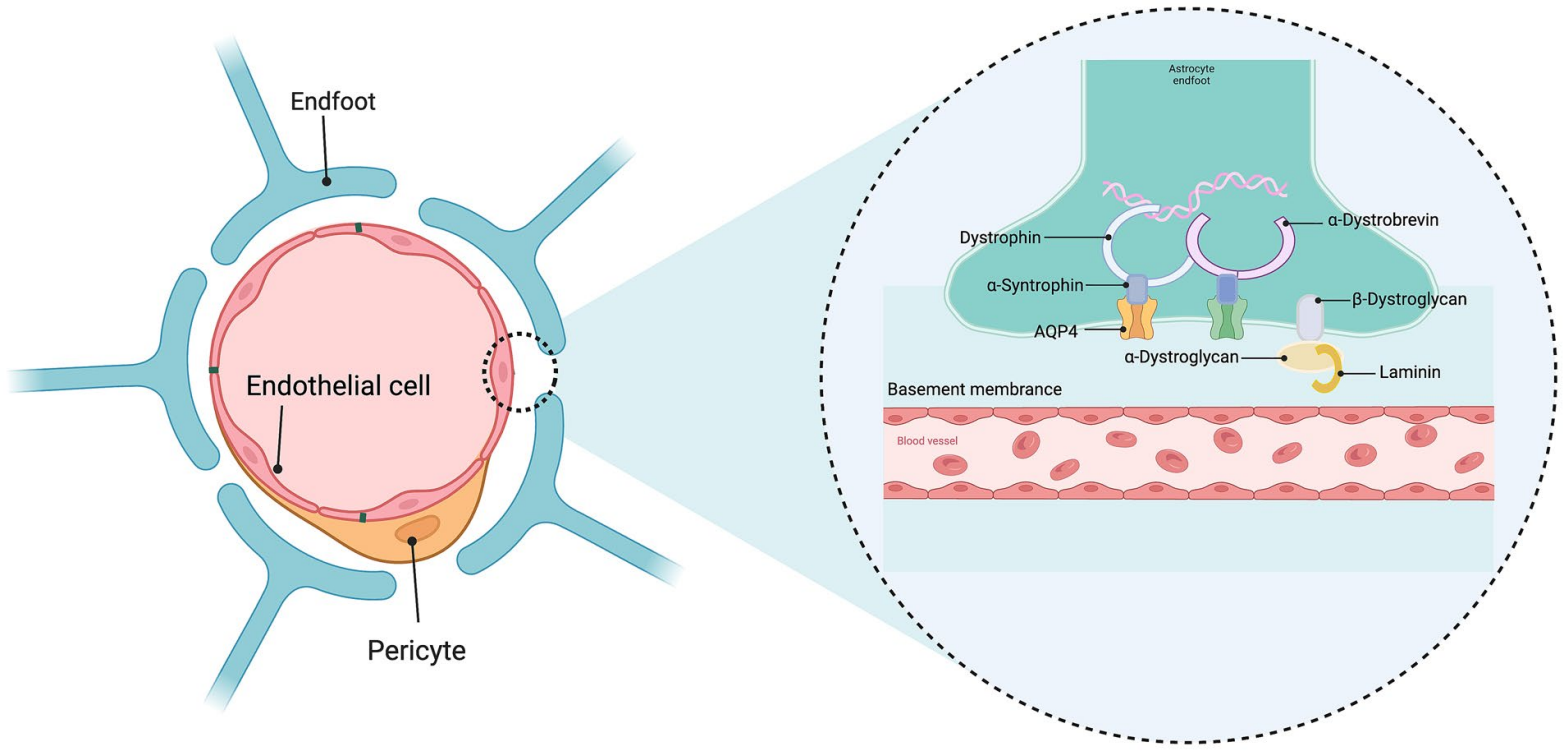
Molecular basis of astrocytic endfoot polarization at the BBB. Endothelial cells and pericytes form the core of the vascular wall, which is surrounded by astrocytic endfeet. On the endfoot membrane, the water channel protein AQP4 is highly enriched and maintains polarized localization under the stabilization of the DAPC, comprising α-syntrophin, dystrophin, α-dystrobrevin, and dystroglycan. Interactions between dystroglycan and laminin in the basement membrane anchor astrocytic membrane domains to the perivascular extracellular matrix, preserving endfoot polarity and providing a structural foundation for the astrocyte–BBB axis (created with BioRender.com).

Aquaporin-4 (AQP4) is the prototypical polarized protein in astrocytic endfeet. Under physiological conditions, AQP4 aggregates into highly ordered orthogonal arrays of particles (OAPs) and is firmly anchored to the perivascular membrane domain of endfeet through the dystrophin-associated protein complex (DAPC) (30–32). In addition to AQP4, the inwardly rectifying potassium channel Kir4.1 is another important molecular marker of endfoot polarization. Kir4.1 colocalizes with AQP4 and jointly contributes to local water and ion homeostasis as well as BBB microenvironmental stability (33, 34). The perivascular localization of AQP4 and Kir4.1 depends on the DAPC scaffold, which connects to basement membrane components such as laminin α2 through the transmembrane protein dystroglycan (DG). This arrangement firmly tethers membrane proteins to the vascular membrane domain of astrocytic endfeet and maintains the polarized architecture required for astrocyte–BBB interactions (30, 33, 35) (Figure 2).

Notably, endfoot polarization is not a static architecture but is dynamically regulated during development and in response to changes in the microenvironment. Studies have shown that AQP4 can rapidly redistribute between intracellular vesicles and the plasma membrane, and that the spatial localization of endfoot-associated proteins can be remodeled after local injury or developmental perturbation (36, 37). This plasticity enables astrocytes to adapt to changes at the neurovascular interface, but also suggests that, under inflammatory conditions or chronic exposure to vascular risk factors, loss of endfoot polarity may represent an early event in the disruption of BBB homeostasis.

### 2.3 Functional mechanisms by which astrocytes maintain BBB homeostasis

#### 2.3.1 Regulation of water and ion homeostasis

Water and ion homeostasis is a key mechanism by which astrocytes maintain BBB homeostasis and NVU function. This process primarily depends on the polarized distribution and functional coupling of AQP4 and Kir4.1 at the astrocytic endfoot membrane. Kir4.1 mediates the uptake and buffering of extracellular K^+^, thereby limiting abnormal local K^+^ accumulation after neuronal activity (38). AQP4 facilitates transmembrane water transport along osmotic gradients and, through functional coupling with Kir4.1, contributes to perivascular osmotic regulation and the maintenance of water and ion homeostasis (39). Thus, the functional coupling of AQP4 and Kir4.1 is not only a structural feature of endfoot polarization but also a critical basis for astrocytes to preserve the BBB microenvironment and prevent water–ion homeostatic imbalance.

#### 2.3.2 Support of endothelial junctions and basement membrane homeostasis

Astrocytic support of the BBB depends not only on structural coverage by endfeet but also on paracrine signaling, basement membrane regulation, and metabolic support. Astrocytes can release soluble factors, including insulin-like growth factors, Wnt growth factors, and glial cell line–derived neurotrophic factor, to regulate the expression and function of tight junction proteins (23). Under injury or ischemic conditions, reactive astrocytes exhibit altered expression of molecules such as VEGF and BDNF, which may participate in endothelial tight junction repair and vascular remodeling (40). Astrocyte-derived laminin γ1 (LAMC1) can be deposited in the perivascular basement membrane and inhibit the degradation of endothelial tight junction proteins (41). Astrocytes can also enhance endothelial energy metabolism and antioxidant capacity through extracellular vesicle–mediated mitochondrial transfer (42, 43). Together, these mechanisms indicate that astrocyte–BBB crosstalk depends not only on direct structural contact but also on the dynamic regulation of endothelial cells and the basement membrane by astrocytes.

#### 2.3.3 Regulation of inflammatory homeostasis

Astrocytes maintain inflammatory homeostasis at the BBB by sensing changes in the perivascular microenvironment and regulating the release of inflammatory mediators. Under chronic stress conditions, astrocytic inflammatory responses cannot be simply classified as “pro-inflammatory” or “anti-inflammatory”; rather, they are highly time-dependent and context-specific. Studies have shown that NF-κB–related signaling in astrocytes changes dynamically in response to stress events, contributing not only to the initiation of inflammation but also potentially to the restriction of inflammatory spread (44). In addition, astrocytes can regulate the migration and activity of microglia, macrophages, and peripheral immune cells through the secretion of cytokines and chemokines (45). Thus, astrocytes help maintain perivascular inflammatory homeostasis at the BBB through reactive state transitions and paracrine signaling.

#### 2.3.4 Perivascular fluid flow and solute clearance

**Figure 3:**
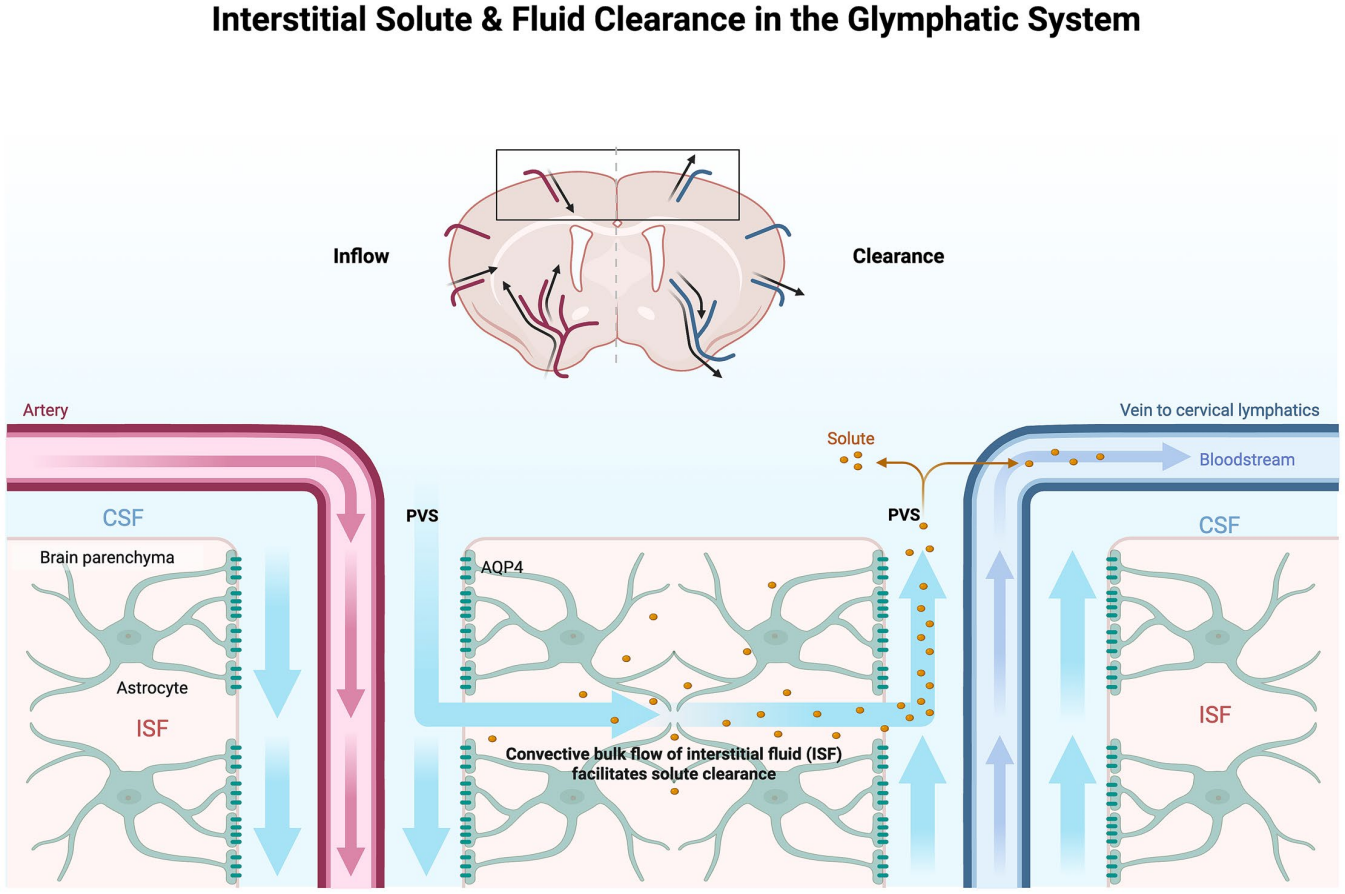
Astrocyte-dependent glymphatic transport and perivascular solute clearance pathways. CSF enters the brain parenchyma via the PVS and exchanges with ISF under the participation of astrocytic endfeet enriched in AQP4, driving convective fluid flow and transport of soluble metabolic waste within the parenchyma. Subsequently, solutes in the interstitial fluid are cleared along perivenous spaces and further drained into the venous circulation and cervical lymphatic pathways. This CSF–ISF exchange system, centered on perivascular pathways and astrocytic endfeet, provides a critical physiological basis for maintaining cerebral fluid homeostasis and metabolic waste clearance (created with BioRender.com).

Astrocytes regulate perivascular fluid exchange and metabolic waste clearance through AQP4 polarization, representing an important mechanism for maintaining BBB homeostasis and cerebral metabolic balance. Cerebrospinal fluid (CSF) enters the brain parenchyma along perivascular spaces (PVS), exchanges with interstitial fluid (ISF), and subsequently drains through perivenous pathways, forming the glymphatic system (GS) and thereby facilitating metabolic waste clearance (46, 47) (Figure 3). This process is jointly regulated by vascular pulsatility, circadian rhythms, vasomotion, and other factors (46–48). AQP4 polarization helps sustain CSF–ISF exchange and the clearance of macromolecular solutes, whereas impaired polarization reduces the efficiency of macromolecular waste removal (49). Therefore, astrocytic AQP4 polarization may provide a mechanistic basis for understanding PVS enlargement and impaired clearance in CSVD. It is important to note that the glymphatic concept, while widely adopted, remains debated. Key unresolved questions include the precise driving forces of perivascular fluid movement, the extent to which AQP4 polarization is required for solute clearance, and the degree to which findings from rodent models translate to the human brain. Furthermore, current imaging surrogates such as DTI-ALPS and EPVS provide indirect measures of glymphatic function and should be interpreted with caution.

#### 2.3.5 Neurovascular coupling and local blood flow regulation

Astrocytes serve as a key hub within the NVU, linking neuronal activity to the regulation of local cerebral blood flow. Through direct contact between their endfeet and the vascular wall, astrocytes integrate neurotransmitter, ionic, and metabolic signals and translate neuronal activity into vasomotor responses (50). The classical model suggests that neuronal activity induces increases in astrocytic Ca^2+^, promoting the release of arachidonic acid metabolites, including prostaglandin E2 (PGE2), epoxyeicosatrienoic acids (EETs), and 20-hydroxyeicosatetraenoic acid (20-HETE), as well as other vasoactive mediators, thereby regulating cerebral small-vessel tone according to local metabolic demands and vascular bed–specific properties (51, 52). However, cerebral blood flow regulation is not entirely dependent on the classical Ca^2+^-mediated pathway. Sensory stimulation–evoked capillary dilation can occur before overt increases in astrocytic Ca^2+^, while elevated cAMP, monoaminergic neurotransmitter signaling, and endfoot ion channel activity may also modulate local blood flow responses (53–55). Thus, astrocytes participate in NVC through both Ca^2+^-dependent and Ca^2+^-independent mechanisms, providing an essential basis for the maintenance of cerebral microcirculation.

While astrocytes clearly influence endothelial function, the reverse signaling–from endothelial cells to astrocytes–is equally important for NVU homeostasis. Endothelial-derived nitric oxide (NO), produced by endothelial nitric oxide synthase (eNOS), not only regulates vascular tone but also modulates astrocytic Ca^2+^ signaling and gap junction coupling. Experimental data suggest that NO can inhibit astrocytic Ca^2+^ oscillations and to regulate the release of vasoactive mediators, thereby fine tuning the neurovascular response (56). Furthermore, factors secreted by the endothelial like endothelin-1 and prostaglandins may affect the activity of astrocytic endfoot ion channels and water transport. In pathological situations such as chronic hypoperfusion, endothelial dysfunction can cause a disruption of this reciprocal signaling and, consequently, disturbed Ca^2+^ dynamics and endfoot polarity without any primary astrocytes damage (50). This crosstalk is bidirectional and highlights the fact that the NVU is an integrated functional unit, such that failures in any part of the unit can be cascaded to other elements by failure of signaling loops. Future research should explore if endothelial factors can induce or worsen dysfunction of astrocytic endfoot in CSVD, thereby providing a potential new target for therapy.

## 3 APOE4-related dysregulation of astrocyte–BBB crosstalk

Apolipoprotein E (APOE), one of the major apolipoproteins in the CNS, is predominantly produced by astrocytes and participates in lipid transport, cell membrane repair, and neuron–glia metabolic coupling (57). Humans carry three major APOE alleles, encoding the APOE2, APOE3, and APOE4 isoforms, among which APOE4 is closely associated with BBB disruption and cerebral microvascular injury (58). Thus, APOE4 may be regarded as an important genetic risk factor linking dysregulated astrocyte–BBB crosstalk to neurovascular uncoupling in CSVD. Before discussing APOE4-associated mechanisms, it is important to contextualize its role in CSVD. APOE4 is unequivocally the strongest genetic risk factor for late-onset Alzheimer’s disease, but its association with sporadic CSVD is more nuanced and population-dependent. Some, but not all, epidemiological studies have linked APOE4 to increased WMH burden and microbleeds, particularly in the context of hypertension or amyloid pathology. The mechanistic studies discussed below–many of which are derived from APOE-transgenic mice or iPSC-derived cells–provide valuable insights into how astrocyte-derived APOE4 may compromise the BBB. However, the extent to which these APOE4-driven pathways generalize to the broader CSVD population, particularly in APOE4-negative individuals, remains an open question.

### 3.1 Astrocyte-derived APOE4 and lipid-driven inflammatory stress

Astrocyte-derived APOE4 disrupts astrocyte–BBB homeostasis by perturbing lipid metabolism. Compared with APOE2/APOE3, APOE4 is more likely to induce lipid metabolic abnormalities in astrocytes, thereby compromising endfoot-mediated maintenance of BBB integrity (58, 59). Before overt vascular injury occurs, APOE4 first remodels astrocytic lipid homeostasis. Abnormal lipid efflux mediated by ATP-binding cassette transporter A1 (ABCA1) is a key component of APOE4-associated lipid metabolic imbalance. When ABCA1 function is impaired, triacylglycerol (TAG)-rich lipid accumulation and aberrant lipid droplet deposition occur in astrocytes, accompanied by mitochondrial stress, increased reactive oxygen species (ROS), and inflammatory responses (59–62).

This lipid-driven inflammatory stress may further weaken astrocytic support of the BBB and NVU. On the one hand, TAG-rich lipid accumulation and increased ROS interfere with astrocytic energy metabolism and membrane homeostasis (60, 61, 63). On the other hand, in the APOE4 context, astrocytes exhibit a more pronounced inflammatory secretory profile, characterized by the upregulation of inflammatory cytokines and chemokines such as CXCL1, CXCL10, and IL-6 (59, 60). In addition, abnormal cholesterol accumulation within lysosomes can impair mitophagy and oxidative phosphorylation, leading to compensatory enhancement of glycolysis and further disrupting astrocytic metabolic support for the BBB (64). Thus, astrocyte-derived APOE4 may drive a pathological cascade of “lipid imbalance–oxidative stress–energy metabolic dysfunction,” thereby establishing an upstream basis for dysregulated astrocyte–BBB crosstalk.

### 3.2 APOE4-related pericyte dysfunction and BBB disruption

Pericytes are embedded within the abluminal basement membrane of endothelial cells and physically interact with astrocytic endfeet, making them essential for maintaining BBB structure and function (65). In the APOE4 context, early BBB injury may manifest as pericyte dysfunction or loss, changes that can precede synaptic abnormalities and behavioral deficits (66, 67).

Pericyte dysfunction can amplify BBB disruption through multiple mechanisms. APOE4 weakens homeostatic signaling between pericytes and astrocytes and induces reactive astrogliosis (68). Reduced pericyte coverage enhances endothelial transcytosis, activates the TGF-*β*/Smad2 pathway, and promotes microglial activation, thereby exacerbating white matter injury and BBB leakage (18). Meanwhile, abnormal pericyte contraction can constrict capillaries through 20-HETE–mediated Ca^2+^ signaling, leading to local cerebral hypoperfusion (69). In damaged pericytes, NADPH oxidase 4 and ROS activate the NF-κB–MMP9 pathway, degrading the vascular basement membrane and tight junction proteins and causing structural barrier disruption (70).

**Figure 4:**
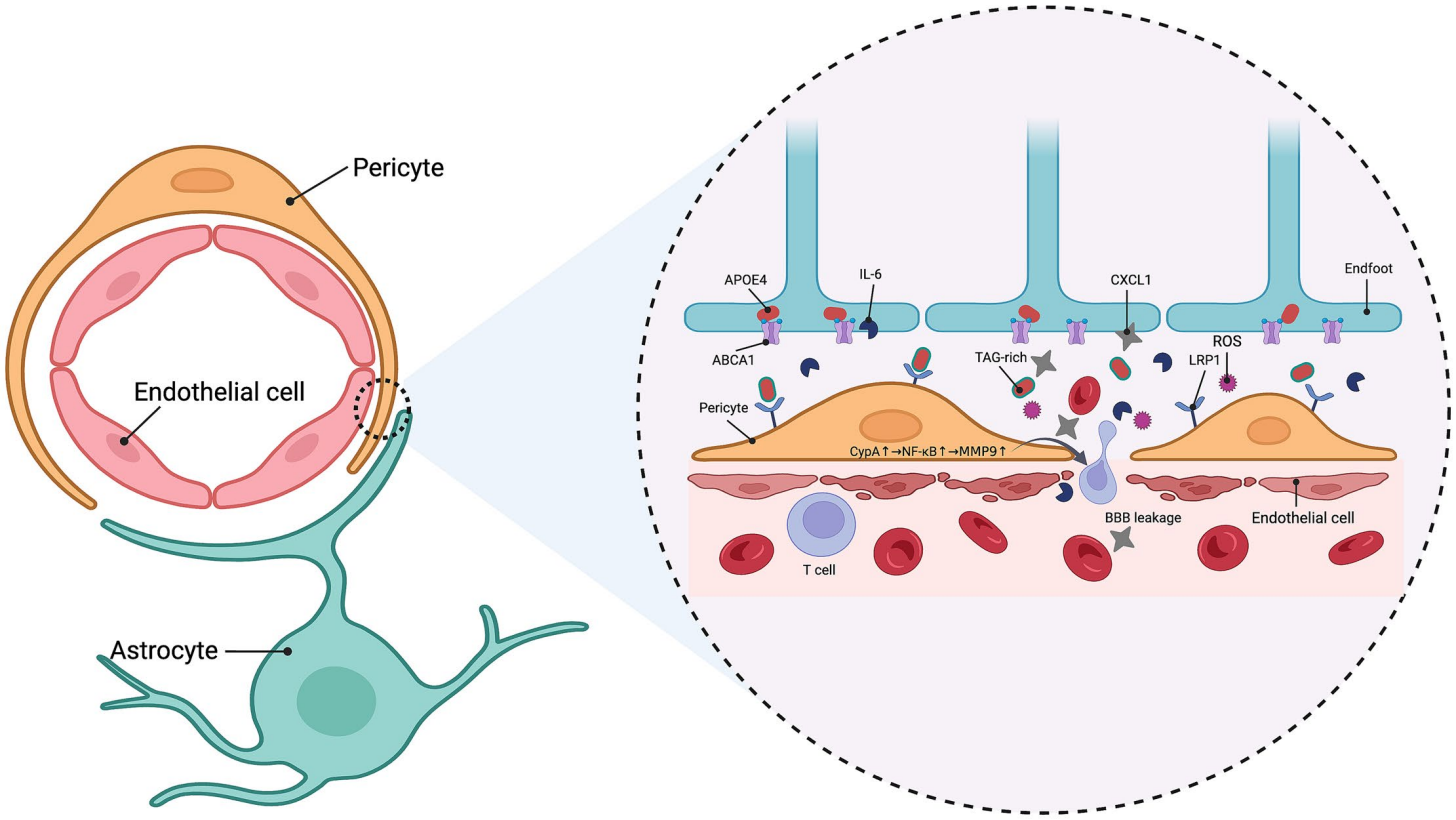
APOE4–driven disruption of astrocyte–BBB crosstalk: linking lipid dysregulation to vascular injury. This schematic illustrates a proposed mechanism by which astrocyte-derived APOE4 compromises BBB integrity in cerebral small vessel disease. First, APOE4 impairs ABCA1–dependent lipidation in astrocytes, leading to accumulation of TAG–rich lipid droplets, oxidative stress, and increased release of inflammatory mediators. These alterations reduce the capacity of astrocytes to support BBB homeostasis. Second, APOE4–related astrocytic dysfunction destabilizes the gliovascular interface and promotes pericyte dysfunction, thereby weakening vascular support and increasing barrier vulnerability. Third, attenuation of protective APOE4–LRP1 signaling activates the CypA–NF–κB–MMP9 pathway in pericytes. This cascade promotes extracellular matrix degradation, endothelial injury, and increased BBB permeability (created with BioRender.com).

Under physiological conditions, APOE binds to low-density lipoprotein receptor–related protein 1 (LRP1) on pericytes and suppresses the CypA–NF-κB–MMP9 pathway. However, in the APOE4 context, this LRP1-dependent protective signaling is attenuated, leading to aberrant activation of CypA in pericytes and subsequent upregulation of MMP9 expression through NF-κB signaling. MMP9 then acts on adjacent endothelial cells and the vascular basement membrane, degrading tight junction proteins and extracellular matrix components, increasing BBB permeability, and promoting plasma protein extravasation, vasogenic injury, and neuroinflammation (71) (Figure 4).

Sustained activation of this pathway further promotes vascular wall remodeling, basement membrane disruption, and reduced vascular reactivity, thereby impairing CSF–ISF solute exchange and waste clearance (72). These alterations can lead to chronic cerebral hypoperfusion, enlarged perivascular spaces (EPVS), and ischemic white matter injury, and are closely associated with CSVD neuroimaging markers such as WMH, lacunes, and EPVS (72, 73). In addition, pericyte dysfunction is linked to loss of AQP4 polarity, impaired perivascular clearance, and reduced cerebrovascular reactivity (74, 75). Therefore, APOE4-related pericyte dysfunction and activation of the LRP1/CypA–NF-κB–MMP9 pathway represent a key molecular route through which APOE4-associated dysregulation of astrocyte–BBB crosstalk is translated into CSVD-related microvascular pathology.

### 3.3 APOE2/3-related protective mechanisms

In contrast to APOE4-mediated dysregulation of astrocyte–BBB crosstalk, APOE2 and APOE3 are more likely to support BBB homeostasis, although their protective effects should be interpreted in the context of specific disease phenotypes. Studies in APOE-transgenic mice have shown that tight junction protein expression, astrocytic endfoot coverage of vessels, and BBB integrity are markedly greater in APOE2/3 backgrounds than in APOE4 mice (58). Multi-omics studies also suggest that APOE3 can sustain NVU signaling networks over time, whereas pericyte injury emerges early in the APOE4 context (66).

Mechanistically, astrocyte-derived APOE2 and APOE3 can effectively bind to LRP1 on pericytes, suppress the CypA–NF-κB–MMP9 pathway, and protect the basement membrane and tight junction proteins, whereas APOE4 fails to efficiently inhibit this pathway (71). Metabolically, astrocytes carrying APOE2 or APOE3 generally exhibit more stable cholesterol synthesis and efflux, relatively preserved lysosomal activity, and lower secretion of inflammatory mediators (59) (Table 2). Taken together, the biological significance of APOE4 extends beyond its traditional role as a genetic risk factor for Alzheimer disease (76). Therefore, APOE isoforms may inform risk stratification and neurovascular-protective strategies in CSVD.

**Table 2:** APOE4–driven imbalance in astrocyte–BBB interactions and APOE2/3-associated homeostatic protection.

Category	Feature	APOE4-associated damage	APOE2/3-associated homeostatic protection
Structure	Astrocytic endfoot and pericyte coverage	Reduced coverage of endfeet and pericytes, weakening structural support for the endothelium (58, 66)	Relatively intact endfoot and pericyte coverage; NVU structure well maintained (71)
	Endothelial tight junctions	Downregulation of Claudin-5, Occludin, ZO-1; compromised endothelial barrier integrity (58, 66, 71)	Normal tight junction protein expression; endothelial barrier integrity preserved (58, 71)
	BBB permeability	Increased tracer extravasation and plasma IgG entry into the parenchyma, indicating enhanced BBB leakage (58, 66, 71)	No significant leakage observed in model systems (71)
Molecular pathways	LRP1/CypA–NF-κB–MMP9 pathway	APOE4 fails to bind pericyte LRP1 → aberrant CypA activation → NF-κB-mediated upregulation of MMP9 → degradation of basement membrane and tight junctions (71)	APOE2/3 binds LRP1, suppresses CypA–NF-κB–MMP9 signaling, preserves basement membrane integrity (71)
	Inflammatory response	Enhanced pro-inflammatory signaling; IL-1β stimulates increased IL-6, TNF-α release (59)	APOE3 attenuates inflammatory responses; APOE2 shows low cytokine release (59)
Metabolism and clearance	Lipid/cholesterol metabolism	Impaired cholesterol synthesis/efflux; reduced lysosomal activity; increased 4-HNE and ROS (59–61, 63)	Cholesterol synthesis/efflux and lysosomal function preserved; APOE2 shows stronger lipid homeostasis (59)
	Aβ clearance	Reduced Aβ uptake and clearance (59)	APOE2 maintains moderate clearance; APOE3 stronger Aβ clearance (59)

In combination, the APOE4 paradigm presents a valuable experimental model with which to dissect the molecular pathways from astrocyte dysfunction to BBB injury. The direct effect of APOE4 on sporadic CSVD should not be over-emphasized, however. Further research of CSVD-specific models (such as hypertensive or NOTCH3 mutant mice) on various APOE backgrounds are required to separate APOE4 cerebrovascular effects from the already well-characterized amyloid-related pathology. APOE genotyping will likely be most useful when combined with other genetic and environmental risk factors for clinical risk stratification, rather than relied on alone.

## 4 Astrocyte-driven BBB destabilization and neurovascular failure

CSVD progression is closely associated with disruption of NVU integrity and BBB dysfunction (77). As key cellular components of the NVU, astrocytes maintain BBB homeostasis under physiological conditions. However, under chronic stressors such as hypoperfusion and inflammation, astrocytes may shift toward an injurious phenotype, thereby exacerbating barrier disruption and dysregulation of cerebral microcirculatory control. Thus, dysregulated astrocyte–BBB crosstalk may represent an important pathological mechanism driving the progression of CSVD toward neurovascular failure.

### 4.1 Reactive astrocytes and inflammatory BBB injury

#### 4.1.1 Pro-inflammatory and chemotactic signaling and immune infiltration

Neuroinflammation and BBB injury are key pathological processes in the development and progression of CSVD. The secretion of inflammatory mediators, including IL-6, CXCL-family chemokines, and TNF-*α*, can activate glial cells, trigger inflammatory cascades, and accelerate CSVD progression (78). During this process, astrocytes not only undergo reactive remodeling but also reshape the inflammatory microenvironment of the BBB through interactions with microglia, endothelial cells, and peripheral immune cells (79). Earlier studies often simplified reactive astrocytes into neurotoxic A1 and neuroprotective A2 subtypes; however, current evidence indicates that astrocytic reactive states are highly heterogeneous and plastic, dynamically shifting between pro-inflammatory and protective phenotypes (80). Cytokines such as TNF and interleukins can induce pro-inflammatory responses (81). In mouse models of CSVD, the expression of tight junction proteins and the vascular homeostasis-related factor VEGFA is reduced, whereas inflammatory and immunoregulatory signaling molecules, including IL-6, IL-10, TNF-*α*, and NF-κB, are markedly increased and closely associated with cognitive impairment (82).

Reactive astrocytes can amplify local inflammation into immune-cell infiltration through pro-inflammatory and chemotactic networks. Although direct evidence in CSVD is limited, studies in sepsis-associated encephalopathy have shown that adenosine can rapidly activate astrocytes and promote inflammatory cytokine release (83). This suggests a potential mechanism that may also operate in the early stages of CSVD, but this remains to be directly verified in CSVD models (Hypothesis). A pro-inflammatory feedback loop involving S100A9/TLR4/NF-κB signaling has been characterized in Parkinson’s disease models (84). If activated in the CSVD microenvironment, this pathway could similarly disrupt NVU homeostasis and amplify glial inflammation, but this hypothesis requires direct testing in CSVD-specific models. As inflammation persists, reactive astrocytes further secrete CXCL-family chemokines and adhesion molecules such as ICAM-1 and upregulate interferon regulatory factor 1, leading to the recruitment and activation of peripheral immune cells. In a traumatic brain injury model, reactive astrocytes upregulate IRF-1 and ICAM-1, promoting peripheral immune cell infiltration (85). Whether a similar cascade occurs in CSVD is an important area for future investigation. The CXCL10/CXCR3/cGAS/AIM2 pathway has been shown to exacerbate endothelial pyroptosis in intracerebral hemorrhage (86). Extrapolating from this finding, astrocyte-derived chemokines may contribute to BBB injury in CSVD, though direct evidence is currently lacking. In addition to classical inflammatory effectors, aberrant astrocytic lipid metabolism may exacerbate this inflammatory network. In Alzheimer’s disease, aberrant SOAT1 expression in astrocytes drives lipid droplet accumulation and pro-inflammatory mediator release (87). This lipid-driven inflammatory pathway may also operate in APOE4-related CSVD pathology, but this remains speculative.

#### 4.1.2 Astrocyte-derived mediators of BBB injury

In addition to indirectly damaging the BBB through inflammatory networks, reactive astrocytes can directly disrupt BBB structure and function by releasing endothelial injury mediators. The S100B/RAGE pathway has been shown to induce endothelial glycocalyx shedding in traumatic brain injury (88). By analogy, astrocyte-derived S100B may contribute to BBB injury in CSVD, but direct evidence in the CSVD context is needed. TNF-STAT3 signaling drives astrocytes to secrete α1-antichymotrypsin, disrupting tight junctions in *in vitro* BBB models and Alzheimer’s disease contexts (89). Whether this mechanism is relevant to CSVD remains to be determined. Upregulation of iNOS and excessive NO production has been linked to downregulation of tight junction proteins in ischemic stroke models (56), suggesting a potential pathway through which reactive astrocytes could compromise BBB integrity in CSVD during hypoperfusion. The CCL2-CCR2 axis has been implicated in NO dysregulation and BBB dysfunction in Alzheimer’s disease and stroke models (90). This pathway may also be relevant to CSVD, but CSVD-specific studies are warranted. Therefore, astrocyte-derived endothelial injury mediators may represent proximal effector mechanisms by which reactive astrocytes compromise BBB integrity.

### 4.2 Loss of endfoot polarity and microvascular wall remodeling

#### 4.2.1 Loss of AQP4 and Kir4.1 polarity

Loss of astrocytic endfoot polarity can induce water–ion homeostatic imbalance (20). Under CSVD-related chronic hypoperfusion, AQP4 and Kir4.1 on the perivascular membrane domain of astrocytic endfeet exhibit loss of polarity, weakening the capacity of endfeet to regulate water–ion homeostasis and local osmotic balance (39, 91). This loss of polarity disrupts signaling communication between astrocytes and endothelial cells, leading to abnormal expression or localization of tight junction proteins (92). Notably, endfoot polarity loss may precede changes in BBB permeability and PVS enlargement (93), suggesting that this process may represent an early event in BBB injury in CSVD.

Studies of ischemic brain injury have also shown that, following reactive astrogliosis, disassembly of OAPs and loss of endfoot polarity can be observed in the peri-infarct region (94). Disruption of endothelial tight junction proteins is also closely associated with loss of endfoot AQP4 polarity (95). Collectively, loss of AQP4 and Kir4.1 polarity may promote the progression of structural BBB injury through water–ion homeostatic imbalance and interrupted endfoot–endothelial signaling, thereby providing a pathological basis for subsequent basement membrane remodeling and vascular wall stiffening. Whether this temporal relationship holds in human CSVD remains an important unresolved question. Current evidence is largely derived from animal models, and longitudinal human studies incorporating serial DCE-MRI and AQP4-specific PET tracers, if such tracers become available, are required to address this gap.

#### 4.2.2 Endfoot–basement membrane uncoupling and microvascular stiffening

Degradation of anchoring protein complexes is a major molecular mechanism underlying structural uncoupling between astrocytic endfeet and the basement membrane. Downregulation or structural destabilization of anchoring complexes at the endfoot membrane, particularly disruption of *β*-dystroglycan (β-DG), can weaken the adhesion between astrocytic endfeet and the basement membrane (20, 96). MMP9 is a key effector molecule mediating this process. Activated MMP9 cleaves the extracellular domain of β-DG, thereby disrupting the linkage between the basement membrane and astrocytic endfeet (97). Clinical studies have shown that serum MMP9 levels in patients with CSVD are inversely associated with cognitive function (98).

Endothelial junction disruption and basement membrane remodeling are important pathological hallmarks of CSVD-related BBB injury (99). Endfoot–basement membrane uncoupling promotes basement membrane remodeling and microvascular wall stiffening. Under CSVD-related stress, the TGF-β1/SMAD3/TIMP-3 signaling pathway is activated, inhibiting normal extracellular matrix (ECM) degradation and turnover and inducing vascular wall cells to produce excessive ECM components (100). Downregulation of VEGFA in astrocytes, together with upregulation of SMAD3 in pericytes, further amplifies TGF-related vascular remodeling signals, leading to basement membrane thickening and vascular fibrosis and increasing the risk of white matter injury and cerebral microbleeds (101). Meanwhile, loss of endfoot polarity can weaken structural support at the endfoot–basement membrane interface, resulting in reorganization, thickening, or degradation of basement membrane components such as laminin and collagen IV (2). Reduced LAMC1 function may also accelerate basement membrane and vascular wall remodeling (41). Thus, loss of endfoot polarity promotes BBB structural disruption, microvascular wall stiffening, and white matter injury in CSVD through aberrant gliovascular signaling and structural destabilization of the endfoot–basement membrane interface.

### 4.3 Glymphatic dysfunction and enlarged perivascular spaces

Although an association between glymphatic dysfunction and CSVD pathology is increasingly recognized, the evidence remains largely correlative. Whether impaired glymphatic clearance is a cause or a consequence of CSVD-related microvascular injury, or simply an epiphenomenon, is not yet established. In the following sections, we review the current evidence with these caveats in mind.

#### 4.3.1 Impaired fluid exchange and enlarged perivascular spaces

CSVD-related vascular wall stiffening and chronic hypoperfusion can impede CSF–ISF fluid exchange and metabolic waste clearance. In turn, impaired glymphatic drainage may lead to EPVS and accumulation of metabolic waste, further aggravating white matter injury and cognitive dysfunction (24, 102).

AQP4 polarization facilitates directional glymphatic flow and metabolic waste efflux. An imbalance in the ratio of the M1 and M23 isoforms of AQP4 can lead to OAP disassembly, weaken the polarized distribution of AQP4 at astrocytic endfeet, and disrupt CSF–ISF fluid exchange (94, 103). AQP4-knockout mice exhibit reduced intracerebral fluid exchange and solute clearance (104). Ischemic brain models have also shown that reduced expression of anchoring proteins disrupts endfoot AQP4 polarity and decreases the efficiency of interstitial fluid efflux (91). Aberrant phosphorylation of AQP4 can induce loss of AQP4 polarity and reduce glymphatic clearance of pro-inflammatory mediators (105). Impaired perivascular fluid exchange–potentially arising from loss of endfoot polarity–may contribute to the structural manifestation of EPVS. However, EPVS are not synonymous with BBB disruption; they may better be regarded as an integrated structural phenotype of impaired perivascular fluid homeostasis, altered arterial pulsatility, and local microenvironmental imbalance (106, 107). The occurrence of EPVS together with impaired glymphatic flow in AQP4-knockout mice further supports the importance of endfoot polarity for glymphatic function (108).

Clinical imaging studies indicate that the pathological significance of EPVS varies across brain regions. Basal ganglia and thalamic EPVS are associated with WMH, white matter microstructural injury, and risk of intracerebral hemorrhage, whereas centrum semiovale EPVS show no significant association with white matter interstitial fluid (ISF) content (109, 110). Basal ganglia EPVS may contribute to WMH formation by impairing ISF drainage and are associated with cognitive decline (111, 112). It should be emphasized that EPVS are not equivalent to BBB disruption. Although white matter EPVS are related to local neuroinflammation and glymphatic function, they are not directly associated with increased BBB permeability (106). Therefore, EPVS may be better regarded as a structural phenotype of glymphatic dysfunction and vascular microenvironmental imbalance, rather than a simple surrogate marker of BBB disruption.

#### 4.3.2 Impaired clearance and cognitive decline

In CSVD, loss of astrocytic endfoot polarity and EPVS impair perivascular fluid dynamics and reduce metabolic waste clearance, representing a potential link through which vascular pathology is translated into cognitive impairment. Clinical imaging studies have shown that patients with CSVD exhibit a reduced diffusion tensor imaging analysis along the perivascular space (DTI-ALPS) index, indirectly indicating impaired glymphatic clearance, which is associated with increased WMH volume, white matter microstructural disruption, and cognitive decline (112–114). Meanwhile, basal ganglia EPVS may contribute to WMH formation by impairing ISF drainage and may enhance the prediction of cognitive impairment risk (111). In addition, glymphatic dysfunction may further amplify cognitive impairment through the retention of metabolically toxic substances such as amyloid-*β* (Aβ) (115). Therefore, glymphatic dysfunction exacerbates CSVD-related vascular pathology, white matter injury, and cognitive decline through ISF retention, WMH progression, and impaired clearance of toxic metabolites. Multimodal imaging combining DCE-MRI (permeability assessment) and DTI-ALPS (glymphatic function) may help differentiate these processes; however, current techniques remain limited in specificity. Emerging CSF-based tracers and AQP4-targeted imaging approaches may further improve mechanistic resolution.

### 4.4 Neurovascular coupling imbalance and neurovascular failure

#### 4.4.1 Imbalance of Ca^2+^ signaling and vascular signaling

Under CSVD-related stress conditions, loss of endfoot polarity and impaired glymphatic drainage disrupt NVC (13, 14). Reduced cerebral blood flow regulatory capacity further aggravates persistent cerebral hypoperfusion, leading to white matter injury and cognitive dysfunction and accelerating CSVD progression (77).

Abnormal astrocytic Ca^2+^ signaling is an important regulatory mechanism underlying NVC imbalance. Under physiological conditions, astrocytes amplify neuronal activity–evoked functional hyperemia through Ca^2+^-dependent signaling (116). In CSVD, astrocytic Ca^2+^ responses to neuronal excitation are attenuated, accompanied by upregulation of mGluR3 and downregulation of AMPA/NMDA receptors, thereby suppressing functional hyperemia (117, 118). Even when intracellular Ca^2+^ transients are enhanced, their spatial integration and network-level coordination may be impaired, resulting in dysregulated local cerebral blood flow control; this process may be related to excessive activation of the Ca^2+^/calcineurin/NFAT4 pathway (119). Insufficient noradrenergic input can also disrupt the norepinephrine–astrocyte Ca^2+^ axis, manifested by delayed Ca^2+^ activation and reduced vasomotor responses (54, 120). In addition, reduced Ca^2+^ transients and decreased functional connectivity within astrocytic networks are associated with impaired vasomotor responses (121, 122). It is important to acknowledge that the role of astrocytic Ca^2+^ in NVC is not without controversy. Several studies have demonstrated that sensory stimulation-evoked capillary dilation and short-duration functional hyperemia can occur in the absence of detectable increases in astrocytic Ca^2+^ (53), suggesting that Ca^2+^-dependent signaling is not universally required for all vasomotor responses. Astrocytic Ca^2+^ may rather control sustained or large-amplitude blood flow changes, whereas other pathways such as cAMP elevation, noradrenergic pathway activation and local K^+^ gradients may be responsible for rapid or transient blood flow changes (54, 55). Their relative contribution may vary as a function of the vascular bed (arterioles vs. capillaries), stimulus and brain region. These complexities suggest that multiple converging deficits such as disrupted Ca^2+^ signaling, impaired K^+^ clearance, altered neurotransmitter handling, and physical detachment of endfeet, may explain why NVC failure occurs in CSVD and affects the ability of the brain to meet metabolic demand. Future studies should focus on unraveling these pathways in CSVD specific models.

Astrocytes-derived vasoactive signaling may be directly involved in the disruption of microvascular vasomotor regulation, when the signaling becomes imbalanced. Increased oxidative stress and inflammation can disrupt the metabolism of arachidonic acid, increase the amount of vasoconstrictive signals (20-HETE) and decrease the amount of vasodilatory signals (EETs and prostaglandin E2) and produce vascular responses that favor vascular constriction or hyporeactivity (123). Maladaptively regulated ATP/adenosinergic signaling could also lead to maladaptive astrocytic output. Abnormal ATP release from the astrocytes and its conversion to adenosine may intensify pathological signaling via A₂A receptors and is connected to the hippocampal network dysfunction and memory impairment (124). Moreover, decreased Kir4.1-mediated extracellular clearance of K^+^ can also contribute to worsening of microvascular vasomotor dysfunction. Vascular wall responsiveness to ionic signals can be reduced by altered local K^+^ gradients, which can lead to poor and slow microcirculatory regulation (125). Overall, astrocytic vascular signaling imbalance drives the progression of NVC dysfunction toward microcirculatory failure in CSVD.

#### 4.4.2 Physical detachment of astrocytic endfeet from the vascular wall and neurovascular failure

**Figure 5:**
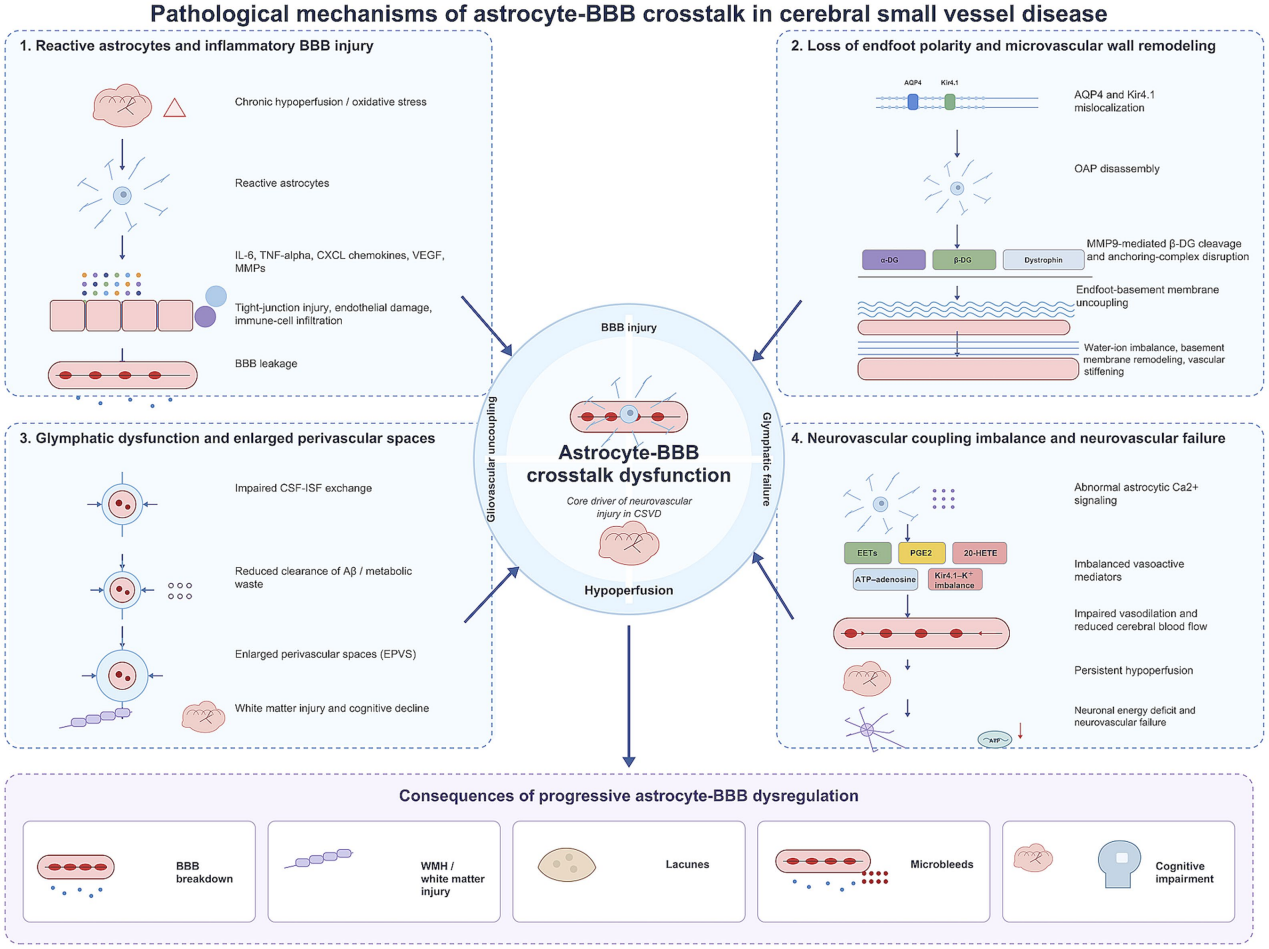
Astrocyte-driven BBB destabilization in CSVD. Chronic hypoperfusion and oxidative stress trigger reactive astrogliosis, inflammatory barrier injury, loss of endfoot polarity, glymphatic failure, and neurovascular uncoupling. These convergent gliovascular cascades promote BBB breakdown, white matter injury, lacunes, microbleeds, and cognitive decline.

Persistent abnormalities in Ca^2+^ signaling and imbalanced vasoactive mediator output jointly contribute to neurovascular dysfunction. Through close contact with cerebral vessel walls, astrocytic endfeet sense local metabolic demands and regulate cerebral blood flow responses (50). Under pathological conditions, reactive astrocytes exhibit dysregulated Ca^2+^ signaling and loss of endfoot polarity. Aberrant Ca^2+^ signaling can drive actin cytoskeletal remodeling in astrocytic endfeet through myosin light chain kinase-related pathways, leading to physical detachment of endfeet from the vascular wall (121). This detachment is accompanied by pericapillary edema, abnormal vesicular trafficking, and ultrastructural BBB disruption, and is associated with vascular cognitive impairment (126). Therefore, the progression from abnormal Ca^2+^ signaling to imbalanced vasoactive mediator output and, ultimately, physical detachment of astrocytic endfeet from the vascular wall may collectively drive the transition from BBB dysfunction to neurovascular failure in CSVD (Figure 5). Available evidence suggests a bidirectional relationship–initial signaling abnormalities may promote detachment, which then amplifies barrier failure (121, 126). Distinguishing cause from consequence requires time–series experiments with longitudinal imaging of endfoot–vascular apposition.

## 5 Regulation of fluid dynamics and lifestyle interventions

Physiological pulsatility and the “valve-like” function of astrocytic endfeet jointly drive fluid dynamics within the GS. Lifestyle interventions, including exercise, sleep optimization, and circadian rhythm regulation, may modulate astrocyte–BBB crosstalk by influencing fluid dynamics, thereby slowing the progression of CSVD-related neurovascular dysfunction.

### 5.1 Physiological rhythms and endfoot-mediated fluid dynamics

CSF flow is jointly driven by cardiac pulsatility, respiration, and spontaneous vasomotion. Arterial wall oscillations generated by cardiac pulsation propel CSF toward the PVS, whereas respiration-related pressure fluctuations facilitate CSF and venous outflow. Spontaneous vasomotion, regulated by rhythmic norepinephrine (NE) release or synchronized neuronal activity, further enhances CSF influx into the perivascular space surrounding astrocytic endfeet (127). Sleep, particularly non-rapid eye movement (NREM) sleep, further strengthens this rhythmic flow (127–129). During NREM sleep, synchronized activity of cortical and hippocampal neurons, fluid and ionic waves, and rhythmic NE release from the locus coeruleus jointly entrain vasomotion with neuronal activity (127). In addition, noninvasive multisensory stimulation can enhance neuronal synchrony and cerebral arterial pulsatility and improve CSF–ISF exchange efficiency by promoting AQP4 polarization (130).

Although the glymphatic system in the CNS lacks anatomical valve structures, directional CSF flow along the PVS can still be observed (131). This suggests that astrocytic endfeet may function as a “valve-like” structure, converting rhythmic pressure fluctuations generated by cardiac pulsatility, respiration, and vasomotion into directed fluid flow. Astrocytic endfeet form the physical boundary of the PVS, and their geometric architecture and mechanical properties confer a valve-like function that channels physiological pulsations into directional CSF movement while limiting retrograde flow (131, 132). AQP4 polarization is crucial in this process. Loss or mislocalization of AQP4 reduces the efficiency of CSF–ISF exchange and may be associated with the formation of EPVS (133).

### 5.2 Exercise-mediated regulation of AQP4 polarization and glymphatic function

Regular exercise may slow CSVD-related neurovascular decline by improving glymphatic fluid flow, AQP4 polarization, and astrocytic inflammatory status. Long-term exercise can enhance fluid movement within the glymphatic system, accelerate metabolic waste clearance, and alleviate neuroinflammation (134). High-intensity interval training may promote the transition of reactive astrocytes toward anti-inflammatory or reparative phenotypes, enhance AQP4 polarization, and improve glymphatic clearance and cognitive performance (135). Exercise can also reduce astrocytic Ca^2+^ overload by inhibiting transient receptor potential vanilloid 4 (TRPV4) channels, thereby restoring AQP4 polarization (136), and can regulate the balance of astrocytic mitochondrial dynamics to limit excessive astrocyte reactivity (137). Animal studies further show that exercise restores the expression of the tight junction protein claudin-5, enhances AQP4–claudin-5 colocalization, and activates the PGC-1α/Nrf1/UCP-2 pathway to promote mitochondrial biogenesis (138). These findings suggest that exercise may preserve astrocyte–BBB crosstalk and delay CSVD progression by improving glymphatic flow and molecular homeostasis (Figure 5).

### 5.3 Circadian rhythms

Sleep and circadian rhythms influence CSVD-related pathological progression by regulating astrocytic states, hemodynamics, and BBB homeostasis. Sleep disturbance is a potential risk factor for cognitive impairment, impaired brain waste clearance, and metabolic dysfunction (139). Sleep deprivation can lead to abnormal expression of clock proteins, impair learning and memory, and alter the expression of circadian clock genes in astrocytes (140, 141). These changes may disrupt ligand–receptor communication between astrocytes and neurons, thereby affecting cognitive function and metabolic homeostasis (139). In addition, circadian rhythms regulate BBB homeostasis by maintaining endothelial tight junctions and NVU function, whereas dysregulated expression of circadian proteins such as BMAL1 can induce neuroinflammation and energy metabolic disturbances and increase BBB permeability (141–143). Sleep deprivation can also activate the NF-κB pathway, increase the secretion of inflammatory mediators, and further disturb BMAL1-related circadian regulation, forming a pathological feedback loop; conversely, sleep improvement may suppress this pathway (144, 145).

Circadian rhythms also regulate glymphatic function and hemodynamics through NE signaling, Ca^2+^ activity, and AQP4 polarization. During NREM sleep, rhythmic NE release from the locus coeruleus drives slow, rhythmic vasomotion (127, 146). NE modulates astrocytic Ca^2+^ activity and influences the release of vasoactive mediators (54). During sleep, astrocytes are partially uncoupled from NE signaling, with reduced Ca^2+^ activity and lower ATP/adenosine levels, thereby favoring glymphatic clearance (146). By contrast, sleep deprivation increases brain NE levels, activates astrocytes, and induces sustained vasoconstriction (147). It also activates the NF-κB pathway through inflammatory mediators such as interleukins and TNF, leading to cerebral hemodynamic abnormalities (84, 145, 147). AQP4 polarization also exhibits circadian rhythmicity. During sleep, AQP4 polarization is enhanced, supporting more efficient glymphatic clearance (148). In APOE4 mice, sleep deprivation and APOE4 synergistically disrupt AQP4 polarization, resulting in impaired glymphatic clearance (149). Sleep improvement can restore the expression of molecular anchoring proteins and AQP4 polarization, thereby promoting metabolic waste clearance and improving cognitive function (150).

## 6 Early diagnosis and reparative strategies

Conventional MRI markers of CSVD, including WMH, lacunes, cerebral microbleeds, and EPVS, primarily reflect structural injury and mid-to-late-stage pathological changes (4). Although these markers have substantial clinical value, their sensitivity for detecting early dysregulation of astrocyte–BBB crosstalk remains limited. Future translational efforts in CSVD should therefore move beyond established structural lesions and further identify early alterations, such as astrocyte dysfunction and BBB microenvironmental imbalance, enabling intervention before the onset of neurovascular failure.

### 6.1 Multimodal biomarkers: identifying early crosstalk dysregulation

Quantitative imaging provides important tools for the early detection of BBB dysfunction. Dynamic contrast-enhanced MRI (DCE-MRI) and metrics such as the BBB water exchange rate (kw) can assess microvascular barrier function more sensitively than conventional structural MRI. Studies have shown that even cognitively normal APOE4 carriers may exhibit widespread cortical increases in BBB permeability, accompanied by elevated free water content and brain microstructural abnormalities (151). The DCE-MRI Ktrans parameter can independently predict cognitive performance and reflect the extent of microstructural injury to the microvascular barrier (152). The APOE4 genotype is associated with reduced kw, suggesting that early functional alterations of the BBB may precede overt structural injury (153). It should be noted that these metrics are not astrocyte-specific biomarkers, but they may serve as indirect imaging evidence of dysregulated astrocyte–BBB crosstalk.

Glymphatic imaging provides a further complementary approach for assessing endfoot polarity, perivascular fluid exchange, and impaired clearance. The diffusion tensor imaging analysis along the perivascular space (DTI-ALPS) index is associated with WMH, EPVS, and cognitive decline in patients with CSVD (154, 155). Multimodal glymphatic MRI composite scores also show potential value in distinguishing CSVD patients with mild cognitive impairment (156). However, EPVS should not be simply equated with BBB disruption; rather, they may be better regarded as an integrated structural phenotype of impaired perivascular fluid homeostasis, altered arterial pulsatility, and imbalance in the local inflammatory microenvironment.

Fluid biomarkers provide a useful complement to imaging-based assessment of astrocyte reactivity and neural injury. Plasma glial fibrillary acidic protein (GFAP) is a potential blood-based biomarker of astrocyte reactivity; elevated levels may indicate enhanced gliotic responses and, to some extent, impaired homeostasis at the astrocyte–BBB interface (157). In sporadic CSVD cohorts, GFAP has been associated with WMH, BBB leakage, and cognitive impairment (158, 159). In addition, neurofilament light chain, CHI3L1/YKL-40, and APOE genotype may be combined with imaging burden, including WMH, lacunes, and PVS, for risk stratification (160, 161). Therefore, future diagnostic strategies should integrate individual markers into multimodal composite biomarker panels, which may facilitate early detection of astrocyte-mediated barrier dysregulation and cerebral microenvironmental disturbance and provide a basis for precision intervention. Plasma GFAP appears closest to clinical implementation, with DCE-MRI Ktrans as a complementary imaging marker. However, both require standardization and validation in large cohorts.

In addition to imaging-based markers, fluid biomarkers offer a complementary and minimally invasive approach to assess astrocyte dysfunction and BBB injury in CSVD. Plasma glial fibrillary acidic protein (GFAP) has emerged as the most promising blood-based marker of astrocyte reactivity. In sporadic CSVD cohorts, elevated plasma GFAP levels have been associated with increased WMH volume, reduced white matter microstructural integrity, and worse cognitive performance (158). Notably, GFAP correlates with DCE-MRI-derived measures of BBB permeability, suggesting that systemic GFAP may reflect, at least in part, barrier dysfunction at the astrocyte–endothelial interface (159). However, GFAP is not specific to CSVD and is also elevated in Alzheimer’s disease, traumatic brain injury, and other neurological conditions. Its utility in CSVD may therefore be greatest when combined with other biomarkers–such as neurofilament light chain (NfL) for axonal injury, YKL-40 for inflammation, and APOE genotype for genetic risk stratification–to form a multi-analyte panel that captures distinct aspects of the disease process (160, 161). Longitudinal studies are now needed to determine whether rising GFAP levels precede or follow the appearance of neuroimaging changes in CSVD, and whether GFAP-guided stratification can improve patient selection for clinical trials.

### 6.2 Reparative strategies targeting astrocyte–BBB crosstalk

At the therapeutic level, the goal should not be to simply suppress or eliminate reactive astrocytes. Instead, functional reprogramming should be pursued to reduce their inflammatory and barrier-disruptive effects while promoting a transition toward homeostatic or repair-associated states.

Metabolic interventions may directly regulate inflammatory transcriptional programs in astrocytes. For example, docosahexaenoic acid (DHA) can inhibit NF-κB activation and reduce astrocytic inflammatory responses (162). Alterations in the gut microbiota may also promote the transition of astrocytes toward a homeostatic state (163). Metabolite-based regulation likewise shows therapeutic potential; the tryptophan metabolite 3-HKA can suppress activation of the AIM2 inflammasome in astrocytes (164). In addition, neuromodulatory approaches, such as transcranial magnetic stimulation with intermittent theta-burst stimulation, can attenuate post-stroke inflammatory responses (165). However, some of this evidence is derived from models of stroke, intracerebral hemorrhage, or other forms of neurological injury, and the long-term efficacy of these strategies in chronic CSVD requires further validation. Although these findings are encouraging, it is important to emphasize that the majority of evidence supporting these metabolic interventions comes from preclinical models of acute injury (stroke, ICH) or other neurodegenerative diseases, rather than chronic CSVD. The long-term efficacy and safety of these strategies in CSVD patients remain to be established in dedicated clinical trials.

Precise restoration of astrocytic endfoot polarity is critical for re-establishing BBB homeostasis. In CSVD, the key issue is not simply an increase or decrease in total AQP4 expression, but rather impaired subcellular localization and polarized distribution of AQP4 at astrocytic endfeet (20, 166). Trifluoperazine can improve AQP4 polarization, restore endfoot morphology, reduce the endfoot-to-capillary area ratio, and enhance basement membrane integrity. It can also increase the expression of endothelial tight junction proteins by inhibiting the MLCK/p-MLC signaling pathway, thereby reinforcing BBB structural stability from both sides of the gliovascular interface (167, 168). Strategies targeting AQP4 should also account for disease-stage differences. During the acute edema phase, inhibition of AQP4 may help alleviate brain edema (169). However, in the chronic phase of microvascular injury, restoring AQP4 polarity may be more beneficial for maintaining BBB integrity and perivascular clearance function (170, 171). Therefore, in translational CSVD therapy, a more rational strategy may be to precisely restore endfoot polarity according to disease stage, rather than applying long-term, nonspecific AQP4 inhibition.

Restoring glymphatic function may also help prevent neurovascular failure and delay CSVD progression. Improving endfoot polarity, modulating the TRPV4–AQP4-related pathway, or enhancing CSF–ISF exchange may facilitate the clearance of metabolic waste, inflammatory mediators, and hematoma-related products, thereby indirectly preserving BBB and NVU homeostasis (171, 172). However, these strategies are currently supported mainly by animal studies or proof-of-concept evidence. Future studies should further validate their efficacy, safety, and optimal therapeutic windows in CSVD-specific models and clinical cohorts.

### 6.3 Stratified interventions: blocking progression toward neurovascular failure

APOE genotype may serve as an important risk-stratification factor for dysregulated astrocyte–BBB crosstalk. Interventions targeting this axis may range from gene editing to nutritional modulation, forming a multilayered framework for precision therapy. Depletion of APOE4 can reduce BBB leakage (173). Inducing conversion of APOE4 toward an APOE2-like phenotype can rapidly improve brain transcriptomic and lipidomic profiles and attenuate astrocytic inflammatory responses (174). Bifunctional liposome-mediated delivery of APOE2 plasmids can reshape the astrocytic APOE phenotype (175). APOE-mimetic peptides, by preserving receptor-binding and lipid-related functions while overcoming limitations in BBB penetration, have shown anti-inflammatory and neuroprotective effects (176). In addition, the effects of dietary fiber and gut microbiota–targeted interventions may be influenced by APOE genotype (177). However, these strategies require clinical validation, and key issues such as delivery methods, long-term safety, and disease-specific indications must be addressed before clinical application.

As a chronic and progressive microvascular disorder, CSVD may offer a window for early identification and stage-specific intervention. In the early disease stage, sustained nutritional or metabolic interventions may improve pathological features. For example, in APOE4 models, long-term DHA supplementation improves memory function and modulates BBB homeostasis (178). Gut–brain axis interventions, such as prebiotic or dietary fiber intake, may enhance astrocytic support of the BBB, although their effects are APOE genotype–dependent (177, 179). In acute or subacute pathological stages, biological therapies such as extracellular vesicles may restore astrocyte function and re-establish the BBB microenvironment (180). However, this evidence is mainly derived from animal models of intracerebral hemorrhage or ischemic injury and cannot be directly extrapolated to the treatment of chronic CSVD. In the chronic progressive stage, exercise, sleep optimization, and management of blood pressure, glucose, and lipids remain foundational strategies (181).

## 7 Limitations and evidence gaps

Some of the existing literature must be acknowledged as having limitations. First, a good portion of the mechanistic framework proposed in this review comes from animal models and *in vitro* systems, and its translatability to human CSVD is unclear. Second, the pathways discussed here (such as astrocytic Ca^2+^ signaling, glymphatic function and endfoot polarity) are mostly associative, and causal relationships are hard to prove in the human brain with current technologies. Third, the field is lacking in standardization of terms like “BBB dysfunction”, “reactive astrocyte states”, and “glymphatic failure” and this makes it difficult to compare between studies. Future studies should focus on multimodal imaging and fluid markers, in longitudinal human cohorts, to establish temporal relationships and causal inferences.

## 8 Conclusions and future directions

CSVD development and progression is a reflection of long-standing gliovascular dysregulation and gliovascular compensatory failure. This review suggests that astrocyte–BBB crosstalk, in particular, may be involved in the progression from BBB dysfunction to neurovascular failure, which contributes towards CSVD progression through inflammatory BBB injury, loss of astrocytic endfoot polarity, impaired GS clearance, and uncoupling of NVC. Additional interventions should be designed to reverse more than one imaging marker or impact more than one pathway. Rather, they should adopt multimodal precision approaches according to the stage of disease, regional heterogeneity and genetic risk with a focus on early blockage of the neurovascular decompensation.

Future investigations are warranted to clarify the causal effect of astrocyte–BBB crosstalk at various phases of CSVD, to better characterize the temporal relationship between BBB leakage, EPVS, and GS dysfunction and to develop composite biomarkers indicating early BBB injury, endfoot polarity loss, and loss of clearance. The incorporation of APOE genotype, vascular risk factors and lifestyle exposures should also be included to define therapeutic targets that can restore endfoot polarity, GS function and NVC homeostasis. These therapies still have a number of challenges to overcome before clinical translation, such as delivery strategies, long–term safety, disease–specific indications and optimal therapeutic windows. Longitudinal cohorts, multimodal imaging, fluid biomarkers and cell–type–specific models will help risk stratification and precision intervention in CSVD. A major challenge for the field is to translate mechanistic observations in animal models to cohorts of humans with CSVD. This will need large, longitudinal, multimodal studies with advanced MRI (DCE-MRI, DTI-ALPS), fluid biomarkers (GFAP, NfL and inflammatory panels), and cognitive assessment to establish temporal relationships, validate biomarkers, and identify optimal therapeutic windows.

Future research needs include focusing on:

(1) Key unanswered questions: Is the loss of AQP4 polarity prior to BBB leakage in human CSVD? What are the temporal relationships between BBB dysfunction, EPVS enlargement and cognitive decline? Is there a way to non-invasively detect astrocytic endfoot detachment?
(2) Biomarkers closest to clinical implementation: Plasma GFAP (astrocyte reactivity), DCE-MRI-derived Ktrans (BBB permeability), and DTI-ALPS (glymphatic function) show particular promise, but need to be standardized and validated in large-scale, multi-center groups.
(3) Experimental models required: CSVD-specific models that more closely mimic human disease, such as aged, hypertensive and NOTCH3-mutant mice with varying APOE backgrounds are required to assess causal relationships and evaluate therapeutic candidates.
(4) Priorities for clinical trials: Clinical trials should start to move beyond single target therapies and explore combination therapies targeting multiple pathways such as restoring endfoot polarity and improving glymphatic clearance. Stratification on the basis of APOE genotype and baseline biomarker levels will be crucial to identify the patients most likely to benefit.
